# NIST Library Identification Probabilities Are the Highest with Cold EI Mass Spectra

**DOI:** 10.1021/jasms.4c00441

**Published:** 2024-12-12

**Authors:** Aviv Amirav, Benny Neumark, Oneg Elkabets, Tal Alon

**Affiliations:** † School of Chemistry, Tel Aviv University, Tel Aviv 6997801, Israel; ‡ Aviv Analytical Ltd., 24 Hanagar Street, Hod Hasharon 4527713, Israel; § Afeka School of Engineering, Tel Aviv 6910717, Israel

## Abstract

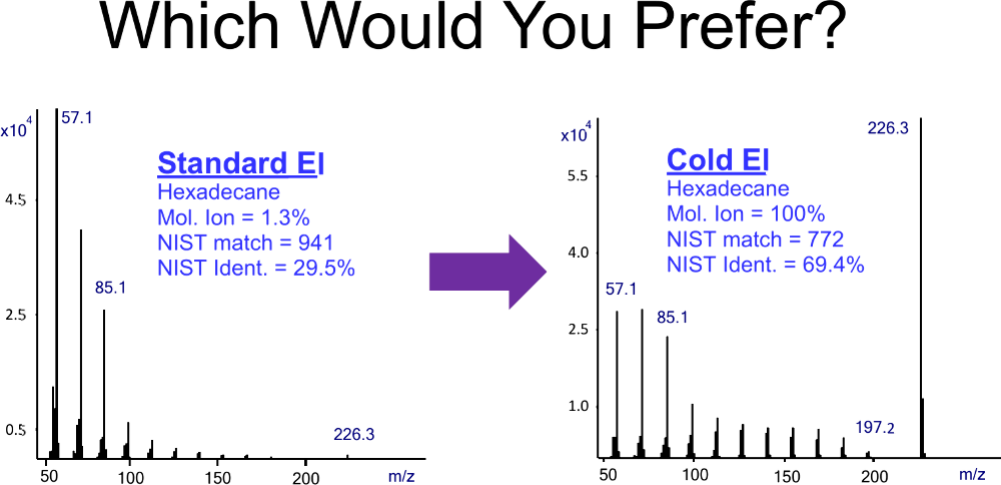

Cold EI improves all of the central GC-MS performance aspects, but even though it is known for providing enhanced molecular ions, it is important to realize that Cold EI mass spectra also include all of the standard EI fragment ions. Thus, Cold EI mass spectra are fully compatible with mass spectral libraries, such as NIST for sample identification. As a result, Cold EI mass spectra (unlike any other soft ionization method) provide highly effective identifications that are often better than those obtained with standard EI for a few reasons:

1). Cold EI mass spectra with enhanced molecular ions typically provide higher identification probabilities than standard EI, even though scoring lower matching factors (false alternatives scores are lowered much more).
2). The visually observed molecular ions further serve to confirm or reject the NIST library identifications.
3). Molecular ions provide isotope abundances that serve with our TAMI software for automatic confirmation or rejection of the NIST library identification and provide an elemental formula in case the compound is not in the library.
4). The Cold EI identification probability can be even higher than that of a mass spectrum with a perfect match of 999 that can be obtained by NIST library searching its own mass spectrum.
5). The clear Cold EI molecular ion enables the use of the NIST library option of constraints search with the molecular ion that further significantly increases the identification probability.

Our findings are demonstrated with mass spectra of hexadecane (n-C_16_H_34_), n-C_40_H_82_, pyrene, nitrobenzene, chlorpromazine, di-n-octyl phthalate, and deltamethrin.

## Introduction

Gas chromatography–mass spectrometry (GC-MS) is widely used for compounds identification. GC-MS usually operates with an electron ionization (EI) ion source, that produces highly fragmented and informative mass spectra.^1,2^ In addition, EI mass spectra (MS) are reproducible, enabling the formation of EI mass spectra libraries such as NIST and Wiley with advanced NIST search and identification algorithms.^3−5^ Having library-based identification with sample compound names and structures, including at the isomer level, is considered one of the most important GC-MS benefits, particularly in comparison with LC-MS. However, for an unknown compound to be truly identified, its mass spectrum must have a molecular ion as it is the most characteristic ion, which also provides the ability to confirm the identification via the provision of the sample compound elemental formula. Based on our analysis of the NIST MS library, we estimate that approximately 30% of the compounds lack the molecular ion or it is weak (below 2% relative abundance), and for compounds with molecular weights above 400 u, roughly 50% of the mass spectra lack a discernible molecular ion. Furthermore, the mass spectra found in the NIST EI library are biased toward some limited enhancement of the molecular ions since they were produced specifically for the library in working conditions that are not used in everyday practice such as using a relatively low ion source temperature that may result in increased ion source related peak tailing.

The NIST MS search software assigns to each search result a match factor and identification probability estimation. The match factor ranges between 0 and 999, and a match factor above 900 is considered excellent, a match factor between 800 and 900 is considered good, and a match factor below that is considered fair or poor. Since the match factor value corresponds to the similarity of the measured mass spectrum to the one found in the library, one may accept an identification as correct if the matching is very high. However, such an approach could be misleading if several high-ranking library candidates have high matching factors. The identification probability serves as a means of reducing false positive identifications by increasing the uniqueness of the query spectrum relative to the best matching wrong library spectra against which it is searched against. The identification probability calculation is more complicated, as it also considers the matching factors of other competing compounds, but it produces much better identification results, particularly if the identification probability of #1 is much higher than of #2. Thus, high identification probability and being at the top of the NIST list with a much higher identification probability than #2 is the best way to ensure proper identification.

GC-MS with Cold EI is based on interfacing the GC and MS with a supersonic molecular beam (SMB) and on sample compound ionization during their flights through a contact-free fly-through ion source for their ionization as vibrationally cold sample compounds in the SMB (hence the name Cold EI). Cold EI improves all the central performance aspects of GC-MS, including the provision of enhanced molecular ions that are compatible with isotope abundance analysis for the provision of an elemental formula. Cold EI mass spectra also retain all of the standard EI fragment ions for having good library-based identification. Furthermore, Cold EI provides an extended range of low-volatility and thermally labile compounds that are amenable for analysis via its possible use of high column flow-rate and a contact-free ion source.

Cold EI was developed by Amirav and his group in 1990,^6,7^ and has been the subject of numerous publications, including reviews,^8,9^ a recent book^10^ and various papers exploring its features and applications.^11−43^

GC-MS with Cold EI typically produces “a lower fit but a better hit” in sample identification, meaning lower matching factors but greater identification probabilities due to the enhancement of the molecular ion and high mass fragment ions.^26^ In ref (26) we discussed and simulated how enhanced molecular ions improve compound Identification by the NIST Library as while the matching factors are reduced with enhanced molecular ions the matching factors of competing compounds are reduced even more, and thus the identification probability is increased within a range of molecular ion enhancement factors. In ref (33) we compared 70 eV electron ionization mass spectra and Cold EI mass spectra of 46 compounds from a few different group types in their NIST library identification probabilities and found that for most of them, the Cold EI identification probabilities were higher than in standard EI. Thus, the claim that Cold EI mass spectra typically provide higher identification probabilities than standard EI mass spectra was properly experimentally demonstrated.

In this paper, we discuss and demonstrate that the Cold EI identification probability can be higher even from that of a standard EI mass spectrum (MS) with a perfect match of 999 that can be obtained by the NIST library searching its own MS. We shall also show that the clear and clean Cold EI molecular ions enable the use of the NIST library option of “constraints search” with the molecular ion that further significantly increases the identification probability. Furthermore, the availability of a clear and abundant molecular ion enables the confirmation or rejection of the library-based identification and the provision of the sample compound elemental formula in case they are not in the library. We shall demonstrate these Cold EI mass spectral features of improved library-based identification in the Cold EI comparison with standard EI mass spectra of seven compounds.

## Experimental Section

We used the 5977-SMB GC-MS with Cold EI, which is based on the combination of an Agilent 7890B GC and a 5977 MSD (Agilent Technologies, Santa Clara, CA, USA) combined with the Aviv Analytical SMB interface and its dual-cage fly through ion source (Aviv Analytical, Hod Hasharon, Israel). In GC-MS with Cold EI, the GC column output is mixed with helium makeup gas for a typical total supersonic nozzle flow rate of ∼50–60 mL/min. The column output is in front of a supersonic nozzle at the end of a temperature-controlled transfer line. Perfluorotributylamine (PFTBA) can be mixed with the makeup helium flow for system tuning and calibration. The sample compounds inside the helium carrier gas expand from a 100 μm diameter supersonic nozzle into a supersonic molecular beam (SMB) vacuum chamber that is differentially pumped by a Varian Navigator 301 turbo molecular pump (Varian Inc. Torino, Italy) with a 250 L/s pumping speed. The SMB, with its vibrationally cold sample molecules, passes through a contact-free fly through dual cage EI ion source.^15^ The ion source filament generates 70 eV ionizing electrons with 6 mA emission current for the ionization of the analytes seeded in the SMB. The ions are focused using two lenses, deflected 90° by a heated ion mirror, and enter the Agilent 5977 MS for mass analysis. The Agilent triple-axis ion detector detects the ions that exit the quadrupole. MassHunter and ChemStation software were used to process and analyze the data.

The Cold EI chromatography separations were performed with a 30 m column with DB5MS-UI film and 1.2 mL/min column flow rate for the analysis of pyrene and di-n-octyl phthalate, while a 15 m column with 0.32 mm I.D. and 0.1 μ DB1HT films and 8 mL/min column flow rate was used for the analysis of hexadecane, n-C_40_H_82,_ nitrobenzene, chlorpromazine, and deltamethrin. The makeup gas flow rate was 46 mL/min in the Cold EI experiments. The 30 m column was selected for a comparative analysis of EPA 8270 semivolatile mixture^44^ while the 15 m column was selected as it provides high flexibility in the GC-MS analysis speed and range of compounds amenable for analysis. The GC oven program typically started at 50 °C, followed by a gradient of 40 °C/min up to 300 °C with an additional 1.75 min hold time for a total analysis time of 8 min. The injection was done using an Agilent split/splitless injector at 260 or 300 °C with a split ratio of 1:9 so that we had 1 ng for each compound on-column (5 ng for n-C_40_H_82_, pyrene, and di-n-octyl phthalate). Experiments with Standard EI were performed with an Agilent 7890 GC and 5977B MSD with its Inert ion source at 280 or 300 °C and 30 m column with DB5MS-UI film and 1.2 mL/min column flow rate and the same on-column sample compounds amounts as in Cold EI. The GC oven temperature program started from 50 °C and increased at 10 °C/min up to 320 °C. We used a NIST library from 2020 version 2.4.

## Results

**Figure 1:**
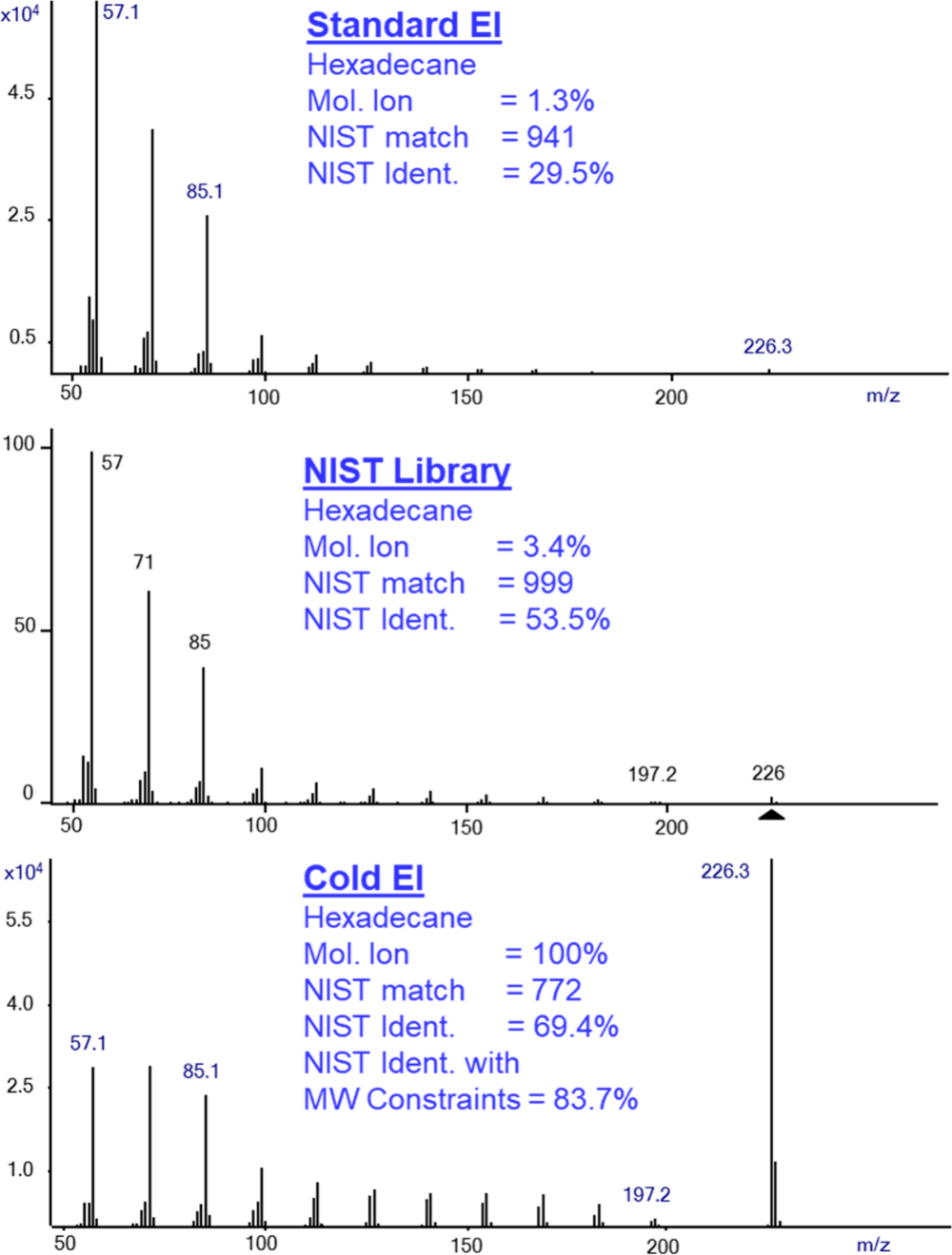
Mass spectra of hexadecane in standard EI (top trace), NIST library mass spectrum (middle trace), and Cold EI (bottom trace). The figure shows the increase of the molecular ion abundance from 1.3% in standard EI to 3.4% in the NIST library and 100% in Cold EI and the corresponding increase of the NIST identification probability from 29.5% to 53.5% in the NIST search of its mass spectrum with perfect match of 999. In the Cold EI mode, the NIST identification probability is further increased to 69.4% and even more to 83.7% while using the molecular ion constraints search.

Figure 1 displays the mass spectra of hexadecane (n-C_16_H_34_) obtained using GC-MS with standard EI (top trace), GC-MS with Cold EI (bottom trace), and the NIST library mass spectrum (middle trace). The figure also shows that the molecular ion abundance in standard EI is 1.3%, the NIST matching factor is 941 and the identification probability is 29.5%. In the middle trace, we show the NIST library mass spectrum of hexadecane, which exhibits 3.4% molecular ion abundance which is 2.6 times greater than in the standard EI mass spectrum. We found that when we put the mouse on the NIST library given mass spectrum, press right click and select library search, we obtain a self-referential library search result, showcasing a perfect match and reverse match factor of 999. The NIST library also provides a given identification probability for this perfect match factor which is typically higher than of the standard EI mass spectra but much lower than 100%. As shown in Figure 1, the identification probability of the NIST library search itself is increased from 29.5% in standard EI only to 53.5% with a perfect match. We note that this search was of a NIST library replica mass spectrum as found by the experimental data. The main library mass spectrum search of itself provides 53.9% identification probability. Figure 1 also demonstrates that the molecular ion abundance is increased to 100% when the Cold EI mode is used, and the NIST identification probability is increased in Cold EI even further to 69.4%. Thus, the Cold EI mass spectrum is fully compatible with the NIST MS search software^3−5^ since as shown, the Cold EI mass spectrum retains all the lower mass fragment ions as in standard EI. In Figure 1 we further include the Cold EI-based NIST library identification while using the molecular weight in the NIST MW constraints search which is 83.7%, thus almost three times higher than the identification probability with standard EI mass spectrum. We can uniquely use this NIST constraints search mode with Cold EI since its molecular ion is not only the dominant most abundant ion, but it is also very clean, with a ratio of >3000 to any higher mass spectral baseline noise.

**Figure 2:**
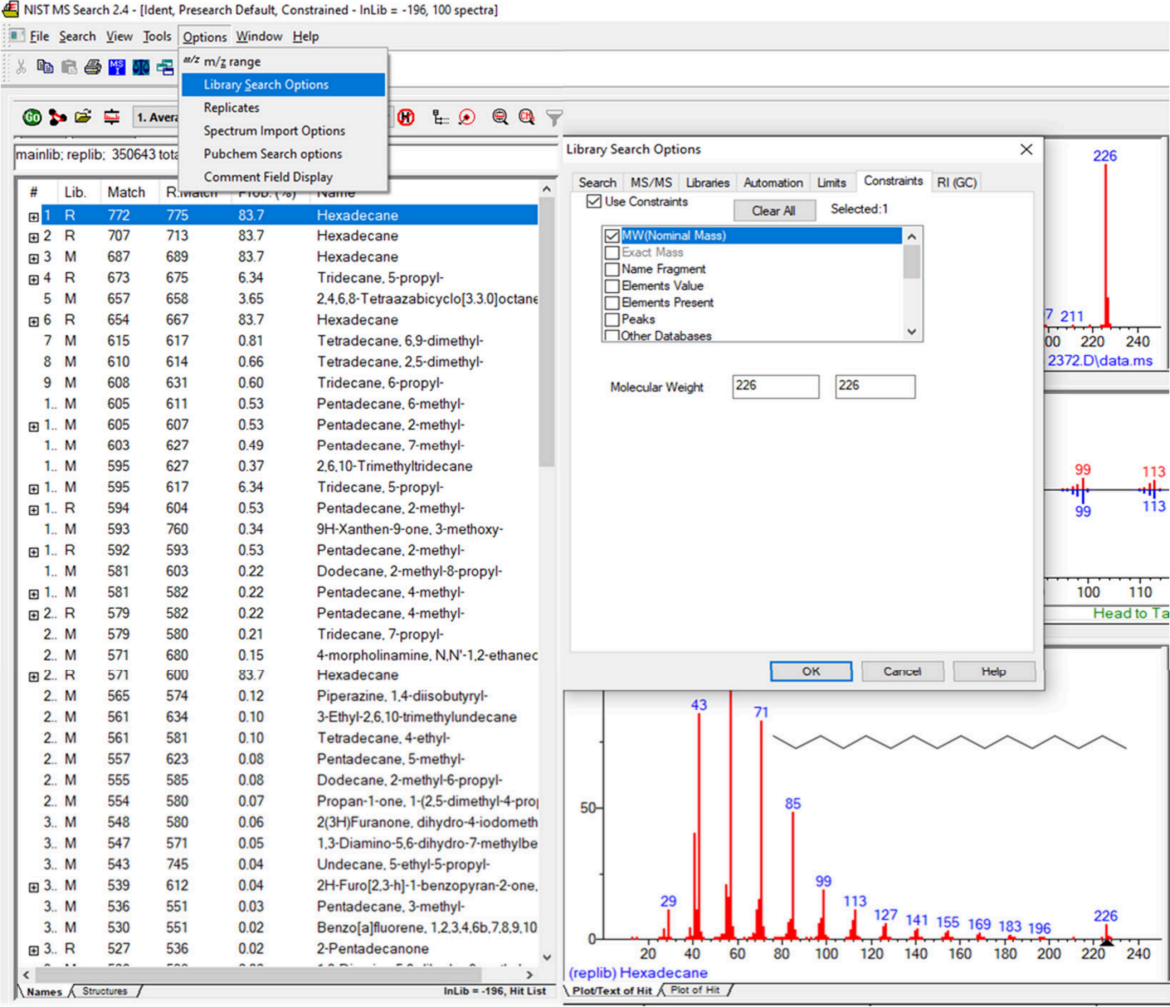
NIST library search screen zoomed at its upper left side. Upon pressing the “options” in the upper left bar we view a subwindow with “library search options” at its second line. Pressing on it brings the “Library Search Options” window, as shown at the right side of Figure 2. It was filled with a molecular weight of 226 for Figure 1 and V in the “Use Constraints” and then OK, followed by the hexadecane MS library search.

In Figure 2 we show the NIST library search screen zoomed-in on its upper left side. Upon pressing the “options” in the upper left bar we view a subwindow with “library search options” at its second line. Pressing on it shows the “Library Search Options” window, as shown at the right side of Figure 2. For Figure 1 data, we filled the “molecular weight” subwindows with 226 and V in the “use constraints” and pressed OK. Upon the search of the NIST library with these MW constraints, we obtained the identification probability of 83.7% as indicated in Figure 1 and also as shown in Figure 2.

In Cold EI we have abundant molecular ions that are also clean, with near-zero noise at masses above the molecular ions. Accordingly, with Cold EI we can uniquely use the NIST MW constraints search and have a further increase in the identification probability, which is much higher for hexadecane than with standard EI.

**Figure 3:**
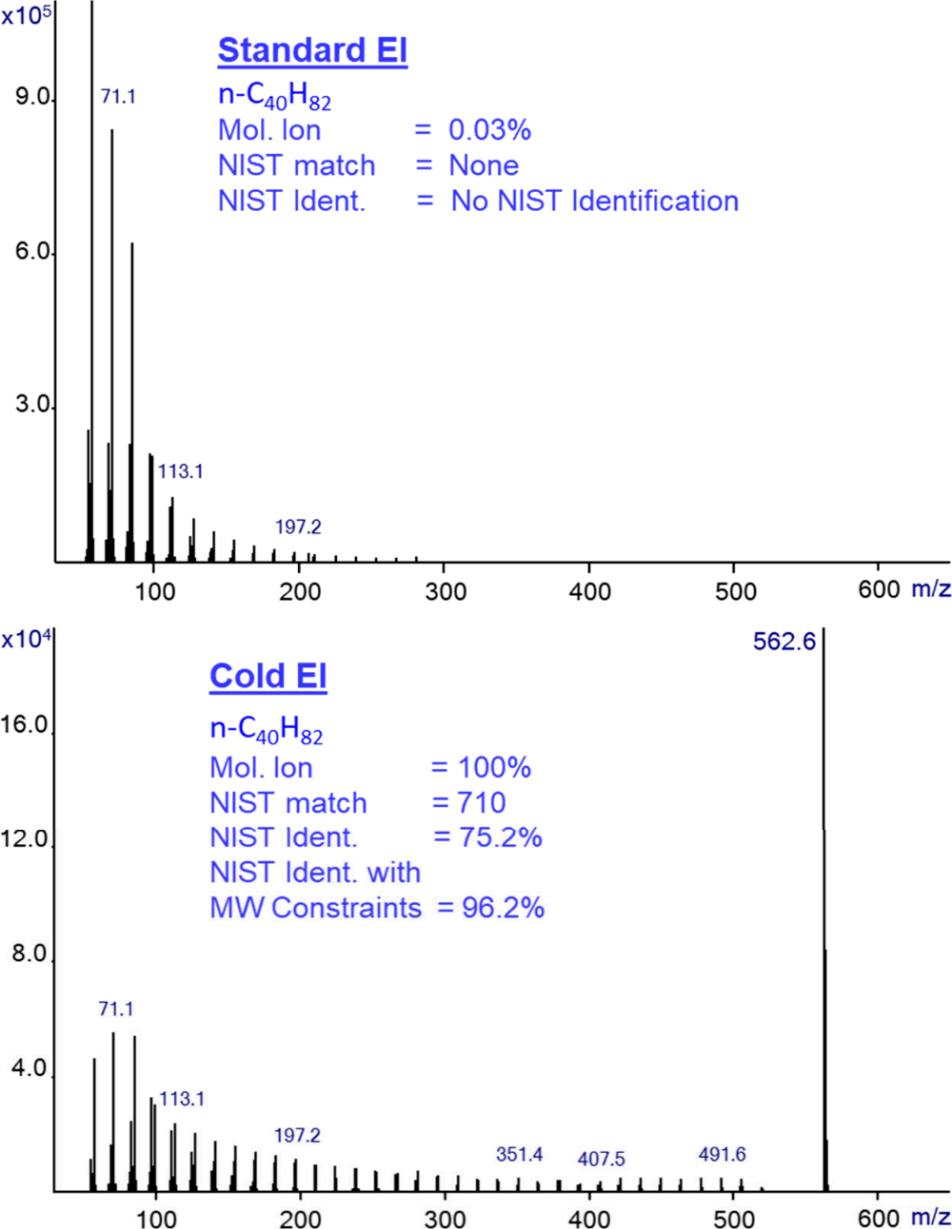
Mass spectra of n-C_40_H_82_ in standard EI mode (upper trace) and Cold EI mode (bottom trace). The molecular ion abundance is increased from 0.03% in standard EI to 100% in Cold EI and the NIST identification probability is increased from “Not Identified” in standard EI to 75.6% in Cold EI and it is further increased to 96.2% upon using the molecular ion constraints in the NIST search.

In Figure 3 we show the standard EI (upper) and Cold EI (bottom) mass spectra that were obtained from the analysis of the relatively large hydrocarbon n-C_40_H_82_. Unlike in Figure 1, the n-C_40_H_82_ NIST library mass spectrum is not shown, as it failed to be identified due to the absence of its molecular ion. Thus, the NIST search itself was also not included as n-C_40_H_82_ failed to be identified. The standard EI molecular ion abundance was measured at a mere 0.03%. In stark contrast, Cold EI yielded a dominant molecular ion (100% abundance), representing an enhancement factor of approximately 10,000. We note that the molecular ion abundance is reduced in standard EI by about 20% per each added carbon atom.^45^ Accordingly, such a reduction in the molecular ion abundance compared with that of hexadecane is not surprising. We found that hydrocarbons with over 22 carbon atoms fail to be identified by the NIST library search of their standard EI mass spectra due to the weakness or absence of their molecular ions, as the fragmentation patterns are very similar to all hydrocarbons. Thus, while all the first hits of the NIST search of n-C_40_H_82_ were with high match factors above 900 the identification failed.

In sharp contrast, the Cold EI search provided a high identification probability of 75.2%, which was further increased to 96.2% upon the use of the NIST MW constraints search.

Figure 3 demonstrates the observation that Cold EI is clearly far better than standard EI in the library identification of large hydrocarbons. Similar data was obtained for dotriacontane (n-C_32_H_66_) as demonstrated and described in our paper on “Cool Classical EI”.^43^ We note that enhanced molecular ions further improve sample identification via the ability to convert the experimental isotope abundances into elemental formulas with our TAMI software.^19,34^ This feature is essential for the provision of elemental formulas for compounds that are not in the library, as experienced in the analysis of novel synthesized organic compounds^24^ and in general unknown analysis. Furthermore, the TAMI software begins with its automatic confirmation or rejection of the NIST library identification based on experimental isotope abundance analysis. In case of rejection, the TAMI software can provide elemental formulas as with high-resolution MS, yet with quadrupole MS.

**Figure 4:**
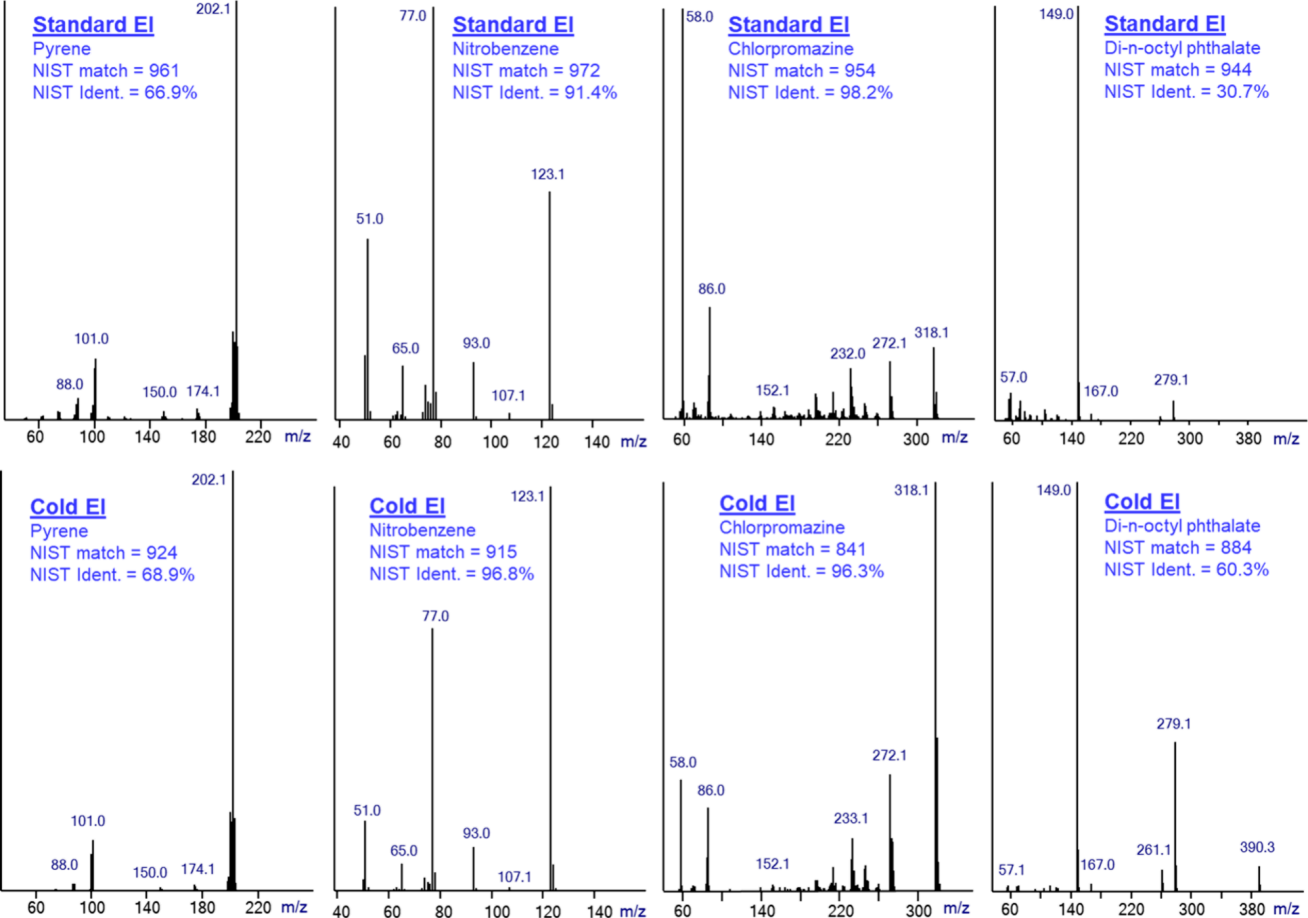
A comparison of mass spectra of pyrene, nitrobenzene, chlorpromazine, and di-n-octyl phthalate (from left to right) in standard EI mode (upper traces) and in Cold EI (bottom traces). The figure includes the NIST identification matches and identification probabilities for these compounds.

In Figures 1 and 3 we explored the NIST library identification of hydrocarbons and compared their Cold EI and standard EI mass spectra. However, in order to learn about the universality of the Cold EI improved NIST identification, we show in Figure 4 a comparison of mass spectra of pyrene, nitrobenzene, chlorpromazine, and di-n-octyl phthalate (from left to right) in standard EI mode (upper traces) and Cold EI mode (bottom traces). These compounds were selected to represent a range of compounds with various degrees of Cold EI molecular ion enhancements. At the left we show pyrene with a dominant molecular ion in both standard EI and Cold EI that has no Cold EI enhancement of its molecular ion. Nitrobenzene is shown on the second left side of Figure 4, and it represents small compounds with available molecular ions. In view of its low number of atoms (14), it has only a small factor of 2.7 Cold EI enhancement of its molecular ion. Chlorpromazine is shown at the second right portion of Figure 4, and it represents medium size compounds with available molecular ions that is enhanced 25 times in Cold EI. Di-n-octyl phthalate is shown at the right side of Figure 4 and it has a very weak (nearly absent, 0.035%) standard EI molecular ion that is enhanced 220 times in Cold EI to 7.7%. We found as shown in Figure 4 that for pyrene both Cold EI and standard EI mass spectra exhibit high match factors and medium-high identification probabilities of 961 and 66.9% for standard EI and 924 and 68.9% for Cold EI. Thus, the NIST identification with Cold EI and standard EI can be considered as similar in view of the visual similarity of their mass spectra and NIST identification data. The identification probabilities of pyrene for its standard EI and Cold EI mass spectra are not high due to the similarity of its mass spectra to those of its isomer fluoranthene. For nitrobenzene, both Cold EI and standard EI mass spectra exhibit high match factors and identification probabilities of 972 and 91.4% for standard EI and 915 and 96.8% for Cold EI. Thus, while the NIST library match of standard EI is higher than that of Cold EI, the small Cold EI enhancement of the molecular ion induces a noticeable increase in the NIST identification probability, which demonstrates the importance of molecular ion enhancement in improving the NIST identification probabilities.

For chlorpromazine, the standard EI mass spectrum exhibits a high match factor and identification probability of 955 and 98.2%, while the Cold EI mass spectrum exhibits a medium-high match of 841 and a high identification probability of 96.3%. Thus, while the 25 times enhancement of the chlorpromazine molecular ion resulted in a reduced match factor, its identification probability remained very high although a little lower than that of standard EI. For di-n-octyl phthalate which serves as an example of a compound with no molecular ion (0.03%), the standard EI mass spectrum exhibits a high match factor of 944 yet with a low identification probability of 30.7%. In contrast, the Cold EI mass spectrum exhibits a medium-high match factor of 884 yet with a much higher identification probability of 60.3% due to its 7.7% molecular ion abundance. Thus, the 220 times enhancement of the di-n-octyl phthalate molecular ion resulted in a much higher (doubled) identification probability than of standard EI. We also note that while the molecular ion on di-n-octyl phthalate was enhanced 220 times, the high mass fragment ion *m*/*z* = 279 was also enhanced over 9 times from 4.4% to 41.3%, and this high mass fragment ion abundance increase also affected the Cold EI match factor and identification probability. Obviously, Cold EI mass spectra are superior to standard EI in the identification of compounds such as large hydrocarbons or di-n-octyl phthalate that have very weak or absent molecular ion, as its availability is essential not only for increased NIST identification probabilities but also for the confirmation of the NIST identifications.

**Figure 5:**
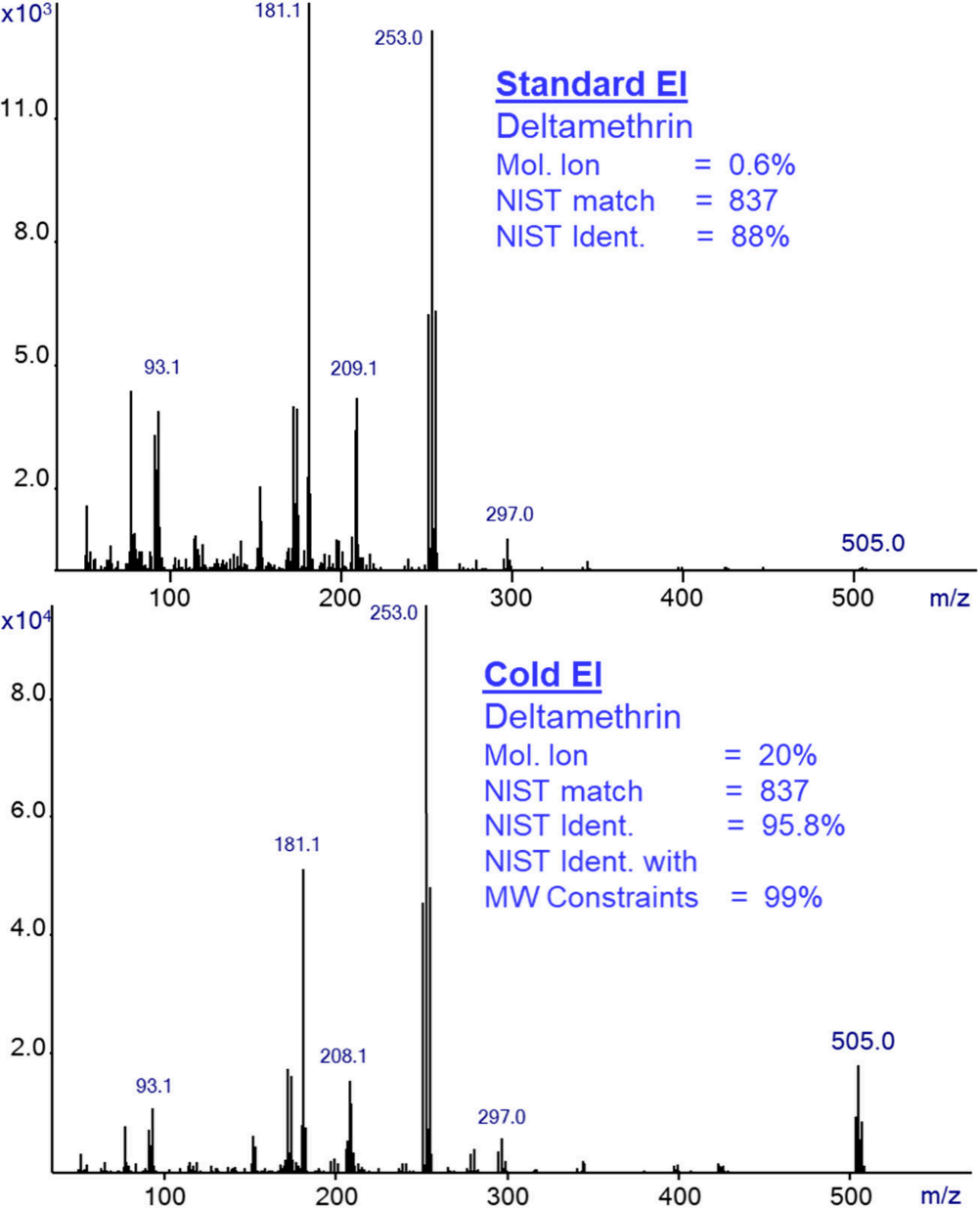
Mass spectra of deltamethrin in standard EI (top trace) and Cold EI (bottom trace). The figure shows the increase of the molecular ion abundance from 0.6% in standard EI to 20% in Cold EI and the corresponding increase of the NIST identification probability from 88% in standard EI to 95.8% in Cold EI and its further increase to 99% while using the molecular ion constraints search in the NIST search.

To further demonstrate the improved NIST identification with Cold EI in comparison with standard EI we show in Figure 5 the identification data for deltamethrin, which is a relatively large and late eluting pesticide. Mass spectra of deltamethrin are shown in standard EI (upper trace) and Cold EI (bottom trace). Figure 5 shows the increase of the molecular ion abundance from 0.6% in standard EI to 20% in Cold EI (*m*/*z* = 505 isotopologue) and the corresponding increase of the NIST identification probability from 88% in standard EI to 95.8% in Cold EI and its further increase to 99% while using the molecular ion “constraints search” in the NIST search. Cold EI can uniquely serve for using the NIST MW constraints search as its molecular ions (all three main isotopologues) have no single ion mass spectral noise above the molecular ions masses, unlike in standard EI in which the weak molecular ions were followed by baseline noise at half the molecular ions intensity. Thus, in Cold EI the molecular ions are clean, clear, and have high confidence. For deltamethrin, the NIST match factors are similar, being 837 after background subtraction in standard EI (1 ng on-column) and 837 in Cold EI without background subtraction that was not needed (1 ng on-column). We also found that the NIST search of itself with a perfect match of 999 resulted in an identification probability of 93%, which is smaller than 95.8% that was obtained from the deltamethrin Cold EI mass spectrum. Thus, deltamethrin provides another clear example of how Cold EI largely improves the NIST identification probabilities while retaining reasonable NIST match factors. A closer look at the deltamethrin mass spectra reveals that in Cold EI the abundance of low-mass fragment ions is decreased but clearly available, while the molecular ions are enhanced with all their isotopologues.

## Conclusions

In this paper, we demonstrated that Cold EI mass spectra with their enhanced molecular ions and availability of all the standard EI fragment ions are fully compatible with mass spectral libraries such as NIST for identification. The NIST library-based identification provides match factors and identification probabilities, but clearly, the identification probability is the most important parameter for trustworthy identification. Cold EI mass spectra (unlike those of any other soft ionization method) provide highly effective identifications that are often better than those obtained with standard EI for a few reasons:

1). Enhanced molecular ions in Cold EI mass spectra typically provide higher identification probabilities than standard EI mass spectra, since the molecular ions are the most sample characteristic ions, and the low mass fragment ions are retained in Cold EI.
2). The visually observed molecular ions further confirm or reject the NIST library identifications.
3). Molecular ions provide experimental isotope abundances that serve with our TAMI software for automatic confirmation or rejection of the NIST library identification and provide elemental formula in case the compound is not in the library.^19,34^ Thus, isotope abundance analysis is an important supplementary method to NIST library-based identification, and it requires having trustworthy molecular ions as provided by Cold EI.
4). We found and described in this paper that the Cold EI identification probability can be higher even than that of a mass spectrum with a perfect match of 999 that can be obtained by NIST library searching of its own MS. This unique observation was described for hexadecane and deltamethrin, and it further demonstrates the importance of having enhanced molecular ions for high identification probabilities.
5). The clear and clean Cold EI molecular ions that have very little or no MS noise at higher masses enable the use of the NIST library option of “constraints search” with the molecular ion that further significantly increases the identification probability, as demonstrated for hexadecane, n-C_40_H_82_ and deltamethrin.

Our findings are demonstrated with mass spectra of hexadecane (n-C_16_H_34_), n-C_40_H_82,_ pyrene, nitrobenzene, chlorpromazine, di-n-octyl phthalate and deltamethrin. We showed that the Cold EI mass spectra are similar to those of standard EI for compounds such as pyrene (and other PAHs compounds) that have dominant molecular ions and a relatively low number of atoms (small internal heat capacity). For small molecules such as nitrobenzene, the enhancement of the molecular ion is small (a factor of 2.7), while it grows to a medium enhancement factor of 25 for chlorpromazine that exhibits medium abundance standard EI molecular ion. On the other hand, compounds such as di-n-octyl phthalate that exhibit a very weak (almost absent) molecular ion exhibit a high Cold EI enhancement factor of the molecular ion (220 for di-n-octyl phthalate). While in this paper we provide data for seven compounds, in ref (33) we compared standard EI and Cold EI mass spectra of 46 compounds from a few different group types in their NIST library identification probabilities and found that for most of them, the Cold EI identification probabilities were higher than in standard EI.

It should be mentioned that even Cold EI can generate in about 1% of the compounds weak or unobserved molecular ions. As an example, aliphatic alcohols exhibit about 1.5% molecular ion for 1-octanol and 5% for 1-octadecanol while small molecules with unstable molecular ions such as CCl_4_ do not exhibit any molecular ion in Cold EI. However, their highest mass fragment with odd *m*/*z* value is clearly a fragment ion. We note that in these cases, the apparent molecular ion mass could be wrong, and the MW constraint should not be used if the molecular ion is not fully trusted as it would give misleading results. We found that Cold EI provides the cleanest and clearest molecular ions among all the ionization methods. As an example, an obvious advantage of electrospray ionization (ESI) is that it provides the precursor mass directly. However, ESI also provides M-1, and adduct ions with Na, K, NH4, as well as dimers, adduct ions and high background ions and thus its provided molecular ion cannot be trusted. Furthermore, many compounds are either weakly or not ionized by ESI. Thus, in many cases the use of ESI needs to be supplemented by negative ions ESI, APCI and APPI and even this combination is weak with hydrocarbons and other small molecules. In the world of GC-MS molecular ions can be provided by chemical ionization (CI) or photoionization (PI) or field ionization (FI) but these ionization methods usually require the replacement of the ion source, are incompatible with NIST library identification and are much less sensitive than standard EI or Cold EI. We note that Cold EI also enables an easy method change to classical EI-SMS or Cool Classical EI modes,^22,43^ but Cold EI is the best ionization method and with it, there is no need for any other ionization mode. Cold EI improves all of the central GC-MS performance aspects, but in this paper, we focused on its provision of enhanced molecular ions combination with full compatibility with NIST library-based identification, unlike with any other soft ionization method. We also demonstrated that its enhanced molecular ions further improve the identification probabilities and trust in the identification.
